# Influence of Soil Phosphate on Rhizobacterial Performance in Affecting Wheat Yield

**DOI:** 10.1007/s00284-024-03685-x

**Published:** 2024-05-11

**Authors:** Gerhardus Breedt, Lise Korsten, Jarishma Keriuscia Gokul

**Affiliations:** 1 Limpopo Department of Agriculture and Rural Development, Towoomba ADC, Private Bag X1615, Bela-Bela, South Africa; 2 Department of Plant and Soil Sciences, University of Pretoria, Private Bag X20, Pretoria, South Africa; 3 Department of Science and Innovation - National Research Foundation Centre of Excellence in Food Security, Pretoria, South Africa; 4 Department of Biochemistry, Genetics and Microbiology, Centre for Microbial Ecology and Genomics, University of Pretoria, Private Bag X20, Pretoria, South Africa

## Abstract

As a primary nutrient in agricultural soils, phosphorus plays a crucial but growth-limiting role for plants due to its complex interactions with various soil elements. This often results in excessive phosphorus fertilizer application, posing concerns for the environment. Agri-research has therefore shifted focus to increase fertilizer-use efficiency and minimize environmental impact by leveraging plant growth-promoting rhizobacteria. This study aimed to evaluate the in-field incremental effect of inorganic phosphate concentration (up to 50 kg/ha/P) on the ability of two rhizobacterial isolates, *Lysinibacillus sphaericus* (T19), *Paenibacillus alvei* (T29), from the previous Breedt et al. (Ann Appl Biol 171:229–236, 2017) study on maize in enhancing the yield of commercially grown Duzi® cultivar wheat. Results obtained from three seasons of field trials revealed a significant relationship between soil phosphate concentration and the isolates’ effectiveness in improving wheat yield. Rhizospheric samples collected at flowering during the third season, specifically to assess phosphatase enzyme activity at the different soil phosphate levels, demonstrated a significant decrease in soil phosphatase activity when the phosphorus rate reached 75% for both isolates. Furthermore, in vitro assessments of inorganic phosphate solubilization by both isolates at five increments of tricalcium phosphate-amended Pikovskaya media found that only isolate T19 was capable of solubilizing tricalcium at concentrations exceeding 3 mg/ml. The current study demonstrates the substantial influence of inorganic phosphate on the performance of individual rhizobacterial isolates, highlighting that this is an essential consideration when optimizing these isolates to increase wheat yield in commercial cultivation.

## Introduction

The remarkable surge in population growth since 1927 can be attributed to our improved understanding of plant nutrition and utilization of mineral fertilizers, resulting in increased agricultural yields per hectare [2]. In light of this, the pressing challenge is to enhance the efficiency of agricultural inputs, while concurrently reducing the adverse environmental impacts of current agricultural practices on non-renewable natural resources [3–6].

Phosphate (P), the second most important primary nutrient essential for various metabolic processes, paradoxically acts as the most limiting nutrient in agriculture [7]. Plants can only absorb free P ions in a monobasic or dibasic form (HPO_4_^−2^ & H_2_PO_4_^−^) but due to the ability of P to absorb, precipitate, and convert to an insoluble form, it ultimately leads to the over application of inorganic phosphate (Pi) fertilizers. This phenomenon explains the substantial P reserves commonly found in agricultural soils [8–11].

With finite P reserves and abundant insoluble P in agricultural soils, Adesemoye and Kloepper [12] suggested a potential solution: the integration of biofertilizers with chemical fertilizers to increase fertilizer-use efficiency. There are three pivotal mechanisms employed by plant growth-promoting rhizobacteria (PGPR) for P solubilization. PGPR can lower soil pH by excreting organic and inorganic acids [13]. Moreover, the release of organic and inorganic acids is associated with the chelation of cations that actively compete with P to prevent complexing within the soil [14]. Lastly, a more soluble form of inorganic P (P*i*) can be released from organic sources by the PGPR-excreted phosphatase enzymes [15]. Under conditions of P scarcity, the phosphate-specific transport system (*PsTS*) regulates phosphate solubilization and transport, inducing two distinct phosphatase enzymes, namely, phosphodiesterase (*PDE*) and phosphomonoesterases (*PME*). *PDE* converts complex organic P to form phosphomonoesters, which are then converted by *PME* to orthophosphate that is easily absorbable [16, 17].

Literature suggests that PGPR can enhance fertilizer-use efficiency in various agricultural crops [18–22]. As observed in previous studies of *Lysinibacillus sphaericus*, and *Paenibacillus alvei* in cucumber, tomato, mung bean, and maize crops, both *L. sphaericus* and *P. alvei* were positive for phosphate solubilization activity [1, 23, 24]. Given the finite reserve of P in soils and its ability to rapidly transform into insoluble forms in the soil, the objective of this study was threefold. We first aimed to evaluate the in-field effect of incremental levels of P on selected PGPR’s ability to promote wheat yield. Secondly, we sought to quantify phosphatase activity in the rhizosphere at the wheat flowering stage. Lastly, we assessed the isolates’ capacity to solubilize incremental levels of insoluble tricalcium phosphate (TCP) in vitro. Altogether, these experiments can provide insight into the potential of these PGPR isolates to make phosphorus more available to plants, which can impact plant growth and yield.

## Materials and Methods

### Field Trial Layout

All field trials were planted at the Towoomba Academic Development Centre (ADC) located on the southern part of the Springbok flats, approximately 4 km southeast of Bela-Bela in the Limpopo Province (28° 21′ E, 24° 25′ S; 1 184 m above sea level). The trials were planted during autumn (April–May) to ensure the onset of vernalisation during winter (June–August) so that sample collection could proceed in June–July annually. According to the 50-year average, the long-term daily average minimum and maximum temperatures at Towoomba ADC vary between 3.0 and 20.8 °C for July and 29.7 °C and 16.5 °C for December, respectively, with an average annual rainfall of 672 mm (Towoomba ADC weather station data). Light frost occurs sporadically during June and July with air temperatures below freezing point for 8 days of the year. The experimental plot consisted of a 2 × 2 m block, with an additional 1.5 m buffer zone around each replicate and was manually planted in the predominantly Huttons ecotope. Each experiment was composed of individual PGPR isolates, either *Lysinibacillus sphaericus* (T19) or *Paenibacillus alvei* (T29), along with an untreated control, applied at five phosphate increments (0%, 25%, 50%, 75% & 100%), and replicated in triplicate in a factorial arrangement completely randomized design (CRD). Fertilizer containing limestone ammonium nitrate (280 g/kg) and superphosphate (10.5%) (Omnia©, Bryanston, South Africa) were applied at planting. Nitrogen levels were maintained at 180 kg/N/ha for all treatments and phosphorus was added as per incremental layout up to 50 kg/P/ha (100%), for irrigated wheat [25]. Each treatment was prepared by homogenously mixing Duzi® wheat cultivar seed (Klein Karoo Seed Marketing, Oudtshoorn, South Africa), one of the dominant cultivars in the region (Agricultural Research Council with 250 g/ha of T19 or T29 inoculated perlite powder (Seeds for Africa©, Big Bay, South Africa). The seeding rate was at a recommended rate of 120 kg/ha and an inter-row spacing of 15cm just before planting. Trials were irrigated bi-weekly to field capacity until physiological maturity after the grain-filling stage. Each subsequent trial was conducted in an untreated field adjacent to the previous season’s trial. Grain yield was collected by destructive harvesting of the entire 2 × 2 m plot.

### PGPR Isolate Maintenance and Treatment Preparation

Isolates of T19 and T29 were retrieved from the University of Pretoria’s PGPR culture collection archive and were maintained using Microbank™ beads (Pro-Lab Diagnostics, Ontario, Canada). Isolates were stored at − 70°C and cultured as needed onto Nutrient Agar® (Biolab, Wadeville, South Africa).

The PGPR treatments used in the field trials were prepared as a powder formulation. Isolates were cultured in sterile Nutrient Broth® (Biolab, Wadeville, South Africa) for 48 h at 25°C in a shaking incubator. Next, 200 g of sterile Perlite® powder was sealed in autoclavable plastic pouches and inoculated with 21 ml of the 48 h-old nutrient broth culture, followed by incubation for 14 days at ambient temperature.

### Phosphate Solubilization

#### Mineral Phosphate Solubilization

In vitro phosphate solubilization (PS) was evaluated following the procedures described by Nautiyal [26] using Pikovskaya-amended medium. The agar medium was prepared by amending Bacteriological Agar® (Biolab, Wadeville, South Africa) with 10 g/l glucose, 5 g/l NH_4_Cl, 1 g/l MgSO_4_.7H_2_O with Ca_3_(PO_4_)_2_ (Merck, Johannesburg, South Africa) at five different concentrations from 0 to 5 mg/ml. Each isolate was stab-inoculated into Pikovskaya-amended media for each increment, and a flame-sterilized inoculation needle was used as a control. Each incremental treatment was replicated five times, and the plates were incubated for 10 days at room temperature. A positive reaction for PS was indicated by the development of a clear halo around the bacterial colony, and the diameter of the halo was measured for the assessment of activity.

#### Field Soil Phosphatase Activity Assay

Soil phosphatase activity was assessed during the third season of the trial at the flowering stage. From each replicate, three rhizospheric soil samples were collected by randomly selecting ten plants per sample and removing the bulk soil from the roots with a sterile spatula. The rhizospheric soil samples were then aseptically removed from the plant root and stored at − 80 °C until use. Modified universal buffer (MUB) was prepared according to Skujins et al. [27] and phosphatase activity was assessed according to Tabatabai and Bremners [28]. Briefly, a standard curve was constructed for the release of *p-*nitrophenol from phosphatase activity, using a range of 0–30 µg/ml at 6 µg/ml increments and color intensity measured at OD_405_. Phosphatase activity was assessed by adding one gram of rhizosphere soil from each sample to 4 ml of MUB, 1 ml of *p*-nitrophenyl phosphate solution (PNP, Merck, Johannesburg, South Africa), and 0.25 ml of Toluene (Merck, Johannesburg, South Africa) before incubating for one hour at 37 °C. The phosphatase activity incubation step was subsequently terminated by the addition of 1 ml of 0.5 M CaCl_2_ (Merck, Johannesburg, South Africa) and 4 ml of 0.5 M NaOH (Merck, Johannesburg, South Africa). The samples were then filtered using Whatman No.1 paper (Merck, Johannesburg, South Africa) and the color intensity at OD_405_ was expressed as µg *p*-nitrophenol g^−1^ soil h^−1^ and compared to the previously constructed standard curve.

### Statistical Analysis

Wheat yield and soil phosphatase activity data were subjected to combined analysis of variance (*df* = 8) to partition variation accounted for by treatment, phosphorus level, and treatment–phosphorus interaction effect using PROC MIXED procedures of SAS (9.4 Statistical Analysis System, North Carolina, U.S.A) at *P* = 0.05. The means were separated using the Tukey test, if significances were observed. Correlation analysis between phosphate and yield and treatment and yield was done using PROC CORR procedures in SAS and the means separated using Spearman’s rank correlation if significances were observed. Phosphate solubilization activity was analyzed using GLM procedures of SAS at a *P* = 0.05 (*df* = 12), and the means were separated using the Dunnett test where significances were observed.

## Results

### Field Trial

In the first season, the results show that isolate T19 significantly (*P* < 0.01) reduced wheat yield by 395.00 kg/ha at 0% P as well as by 146.70 kg/ha and 236.70 kg/ha for P levels 25% and 50%, respectively, when compared to the control yields at the equivalent phosphate levels (Table 1). Treatment T29 also significantly reduced yield by 582.5 kg/ha at the 25% P level and by 91.70 kg/ha at the 0% P level, relative to the respective control yields. Isolate T19 significantly increased wheat yield by 324.36 kg/ha and 909.60 kg/ha at 75% P, and 100% P. A similar increase was noted for isolate T29 at 50% P, 75% P, and 100% P with yields of 4.1 kg/ha, 170.33 kg/ha, and 367.10 kg/ha higher than that of the respective control yields.

**Table 1:** Effect of seed treatment with selected PGPR strains from the University of Pretoria’s PGPR culture collection on wheat yield at various phosphate concentrations over three successive growing seasons

Treatment	% P	Yield (kg/ha)*		
		Season 1	Season 2	Season 3
Control	0	4 205.00 ± 150.33^abcd^	2 855.56 ± 535.46^abc^	4 205.56 ± 195.32^bc^
T19		3 810.00 ± 224.13^ab^	3 679.63 ± 309.04^bcde^	4 538.89 ± 112.57^cd^
T29		4 113.30 ± 61.66^abc^	2 311.11 ± 328.59^a^	3 327.78 ± 89.25^ab^
Control	25	4 352.50 ± 42.13^a^	3 194.47 ± 373.24^abc^	4 383.33 ± 380.33^cd^
T19		4 205.80 ± 13.48^abc^	3 363.89 ± 210.30^abcd^	3 466.67 ± 224.24^ab^
T29		3 770.00 ± 301.47^a^	2 334.26 ± 174.99^ab^	6 055.56 ± 295.08^efg^
Control	50	4 389.20 ± 302.00^bcd^	3 001.85 ± 930.10^abcd^	3 311.11 ± 285.81^ab^
T19		4 152.50 ± 182.77^abc^	3 392.60 ± 202.08^abcd^	5 188.89 ± 211.57^de^
T29		4 393.30 ± 192.16^bcd^	2 357.41 ± 154.42^ab^	5 641.66 ± 368.06^ef^
Control	75	4 175.67 ± 113.31^abc^	3 533.34 ± 510.09^bcd^	3 172.22 ± 85.90^a^
T19		4 500.03 ± 64.91^ cd^	3 998.15 ± 208.36^cde^	3 672.22 ± 303.48^abc^
T29		4 346.00 ± 60.72^abcd^	3 020.37 ± 283.23^abc^	6 677.78 ± 607.42^f^
Control	100	3 944.20 ± 109.38^abc^	4 368.52 ± 238.54^de^	6 138.89 ± 556.45^fg^
T19		4 583.80 ± 274.06^d^	4 775.93 ± 207.62^f^	4 088.89 ± 104.83^abc^
T29		4 311.30 ± 284.06^abcd^	3 444.45 ± 147.22^abcd^	6 483.33 ± 391.67^fg^
Phosphate (*df* = 4)		*P* < 0.001	*P* < 0.001	*P* < 0.001
Treatment (*df* = 2)		*P* < 0.057	*P* = 0.888	*P* < 0.001
Phosphate*treatment (*df* = 8)		*P* < 0.001	*P* < 0.05	*P* < 0.001

*Wheat yield determined at a moisture content of 12%

^a–g^Treatment means within the same column and phosphate level followed by the same letter do not differ significantly (*P* = 0.05) according to the Tukey test

During the second season, only isolate T19 demonstrated an increase in wheat yield at all phosphate levels, with improvements of 479.63 kg/ha, 169.42 kg/ha, 390.75 kg/ha, 464.81 kg/ha, and 407.41 kg/ha for the 0% P, 25% P, 50% P, 75% P, and 100% P levels, respectively, when compared to the controls. Treatment with isolate T29 resulted in reduced yields at all phosphate levels when compared to the respective controls, with a significant (*P* = 0.05) reduction of 860.21 kg/ha and 924.07 kg/ha at 25% P and 100% P, while reducing yield by 544.45 kg/ha, 644.44 kg/ha and 512.97 kg/ha at 0% P, 50% P and 75% P when compared to control yields.

Season three showed that isolate T19 significantly increased wheat yield at 0% P, 50% P, and 75% P by 333.33 kg/ha, 1 877.78 kg/ha, and 500.00 kg/ha. However, the same isolate significantly reduced yield at 25% P and 100% P, by 916.66 kg/ha and 2 050.00 kg/ha, when compared to the respective control yields. Isolate T29 significantly increased yield at all phosphate levels except 0% P and 100% P, with increases of 1 672.23 kg/ha (25% P), 2 330.55 kg/ha (50% P), and 3 505.56 kg/ha (75% P). At 0% P, T29 significantly reduced yield by 877.78 kg/ha but increased yield by 344.44 kg/ha at 100% P, when compared to the control yield of 4 205.56 kg/ha.

The results (Table 1) showed a significant (*P* ≤ 0.05) effect on yield due to the interaction between phosphate and treatment. The individual variable effect on yield results indicated that the treatment effect was only significant (*P* < 0.01) in the third season, while the phosphate effect was significant (*P* < 0.01) for all seasons. The correlation results (Table 2) support this significant interaction results obtained except for the phosphate effect on yield during the third season.

**Table 2:** Spearman’s correlation of wheat yield of treatment (T19 and T29) and incremental levels of inorganic phosphate over three production seasons

	Yield Season 1		Yield Season 2		Yield Season 3	
	*r*—value	*P*—value	*r*—value	*P*—value	*R*—value	*P*—value
Phosphate	0.42	0.004	**0.57**	< 0.001	**0.27**	0.074
Treatment	0.07	0.626	**-0.38**	0.011	0.44	0.003

*Values in bold indicate significance at *P* = 0.05, separating the means using Spearman’s rank co-efficient

### Phosphate Solubilization

Isolate T19 (Table 3) was the only isolate able to solubilize tricalcium phosphate (TCP)-amended Pikovskaya media based on halo size within the concentration range of 3–5 mg/ml. When phosphatase activity at the wheat flowering stage (Table 4) was considered, significant (*P* < 0.001) hydrolysis of* p*-nitrophenol was noted solely for isolate T19 at 25% P, when compared to the control. A significant (*P* < 0.001) reduction in phosphatase concentration was also observed for isolate T29 at all P levels exceeding 25% P, while isolate T19 exhibited this reduction only at 75% P.

**Table 3:** In vitro solubilization of incremental tricalcium phosphate concentrations by selected rhizobacteria on Pikovskaya media

Isolate	Tricalcium phosphate (mg/ml)	Solubilization	Halo diameter (cm)
T19	0	–	0.00^a^
	1	–	0.00^a^
	2	–	0.00^a^
	3	+	1.20^b^
	4	+	1.40^b^
	5	+	1.40^b^
T29	0	–	0.00^a^
	1	–	0.00^a^
	2	–	0.00^a^
	3	–	0.00^a^
	4	–	0.00^a^
	5	–	0.00^a^
Control	0	–	0.00^a^
	1	–	0.00^a^
	2	–	0.00^a^
	3	–	0.00^a^
	4	–	0.00^a^
	5	–	0.00^a^
*P*—value (*df* = 12)			*P* < 0.001
Ms-value			0.526

^a,b^ Treatment means within the same column followed by the same letter do not differ significantly (*P* = 0.050) according to the Dunnett test

**Table 4:** Effect of selected rhizobacteria on soil phosphatase activity at different phosphate levels at wheat flowering

Treatment	Phosphate*	Nitrophenol (µg/g/hr)
Control	P0	11.38^abc^
T19		14.43^cde^
T29		12.84^bcd^
Control	P25%	12.43^abcd^
T19		16.68^ef^
T29		12.55^abcd^
Control	P50%	14.82^cde^
T19		12.48^abcd^
T29		9.08^a^
Control	P75%	19.31^f^
T19		13.00^bcde^
T29		10.16^ab^
Control	P100%	19.25^f^
T19		20.05^f^
T29		12.86^de^
Phosphate (*df* = 4)		*P* < 0.001
Treatment (*df* = 2)		*P* < 0.001
Phosphate*treatment (*df* = 8)		*P* < 0.001

*Fertilizer percentage from the recommended 7t/ha for the Duzi cultivar at 180 kg/N/ha and 50 kg/P/ha

^a^^−^^f^Treatment means within the same column and phosphate level followed by the same letter do not differ significantly (*P* = 0.050) according to the Tukey test

## Discussion

In existing literature, limited multi-seasonal field trial studies focus on the influence of individual PGPR isolates on wheat yield. The wheat yield results represented in this study (Table 1) highlight the significant impact of soil phosphate levels on the ability of individual PGPR isolates to influence wheat yield throughout all seasons of evaluation.

### Field Season Variability in Wheat Yield

The first season showed that application of both isolates resulted in a decline in wheat yield when P levels fell below 50%, however, a significant increase in wheat yield was observed when P levels reached or exceeded 50% P. This resonates with previous research findings on tomato, chilli, and wheat regarding optimal fertilizer-use efficiency using PGPR that suggested a 75% fertilizer level precedes PGPR performance stability deterioration [22, 29, 30], albeit using a PGPR consortium. During the second season, T19 exhibited its capacity to enhance wheat yield, while isolate T29 consistently reduced wheat yield across all levels of P. During the final season, both isolates increased wheat yield above 25% P, except for isolate T19 which demonstrated a significant decrease at 25% P and 100% P. This phenomenon emphasizes the context-dependent nature of PGPR performance, which can vary even among individual isolates.

Two independent multi-seasonal studies conducted by Khalid et al*.* [31] and Oksel et al. [32] found similar wheat yield variability as in the present study. Both studies noted that their respective PGPR treatments increased wheat yield in one season but led to declines during another. However, these studies remained silent on the underlying reasons for these observations, accentuating the intricate nature of plant–microbe interactions that continue to challenge our comprehension [33]. Several factors could potentially contribute to the observed yield variation. Firstly, PGPR-cultivar incompatibility cannot be ruled out, as it has been observed in a soybean study that specific PGPR reduced growth parameters in certain cultivars but not in others [34]. A second, and perhaps more probable reason, is the response of the PGPR to seasonal fluctuations determined by abiotic factors or interaction and competition within the local microbiome which can act deleteriously [35]. The host plant’s response to these external factors could result in the over-production of compounds that limit plant growth, e.g., phytohormones, and phytotoxins. A third reason may lie in the importance of phosphate in PGPRs T19 and T29 metabolic processes, which, under limiting conditions, can initiate PGPR phosphate scavenging mechanisms that produce secondary metabolites which in turn could cause an imbalance in a plants physiological system [36].

### Influence of Phytohormones on PGPR Performance

Plant hormones, which influence almost all physiological plant systems, can be perturbed by microbially excreted phytohormones. While elevated levels of auxin and cytokinin have been associated with pathogenic microorganisms [37], Lobo et al. [33] found that elevated concentrations of PGPR-produced auxin did not negatively influence auxin-sensitive growth parameters in tomato plants. Ethylene, on the other hand, which typically promotes root growth but stunts apical meristem growth, might contribute to the observed yield variations [38]. Salicylic acid, another well-known phytohormone, promotes plant immunity but can also suppress plant growth at elevated concentrations by reducing metabolic energy available for plant growth through interference with the plant metabolic system, by affecting the balance in salicylic acid-related phytohormone systems or by affecting antioxidant gene transcription that regulates reactive oxygen species [39]. Whole-genome sequencing of *Lysinibacillus sphaericus* (T19; accession number SAMN19982556) and *Paenibacillus alvei* (T29; accession number SAMN19982557) revealed the presence of genes associated with the production of salicylic acid (SA), indole-3-acetic acid (IAA), and cytokinin but not genes related to abscisic acid, ethylene, brassinosteroid, jasmonic acid, or strigolactone-producing pathways. As such, we postulate that the seasonal fluctuation in the yield results from T19 and T29 can only be attributed to the isolates interacting with the host plant and the local microbiome, promoting the imbalance of plant physiological systems with a detrimental effect on wheat yield [33].

### Phosphate Solubilization Mechanisms

When phosphate solubilization of P*i* was evaluated in vitro using Pikovskaya media amended with tricalcium phosphate (Table 3), isolate T19 displayed the unique ability to solubilize TCP at concentrations exceeding 3 mg/ml. Notably, the presence of plant growth-promoting genes associated with P*i* solubilization via organic acid production was verified (data not shown) [40] in both isolates, which contradicts the earlier findings by Breedt et al*.* [23]. However, Egelkrout et al*.* [41] and Patterson et al. [42] suggested that the non-transcription/translation of genes could be attributed to various factors if not triggered. Zeng et al. [43] supported this by illustrating that P can trigger phosphate solubilization gene activity, consistent with the present study, indicating that the increase in TCP levels induced PS mechanisms in isolate T19 but not in isolate T29.

### Soil Phosphatase Activity and Phosphonate Transport System Regulon (Pho)

When field phosphatase activity at flowering is considered (Table 4), soil phosphate, treatment, and the interaction between the two have a significant (*P* < 0.01) effect on the soil phosphatase concentration. The lower levels of soil phosphatase were noted at higher percentages of P*i* and vice versa, when compared to the respective controls, except for isolate T19 at 100% P*i*. The lowest level of phosphatase for both isolates was noted at 50% Pi, but a significant difference in phosphatase emerged at 75% P*i* between the isolates and the control treatment. The inverse relationship between soil microbe phosphatase levels and mineral P fertilizer level suggests the inhibition of the phosphonate transport system regulon (*Pho*). The *Pho* regulon only activates P solubilization mechanisms, such as enzyme and organic acid excretion, during P*i* starvation [16, 44, 45]. The lower levels of phosphatase indicate sufficient soil Pi for microorganism utilization, reinforcing our finding and that of various other field trials indicating an optimum percentage of P is essential to promote plant growth and yield.

## Conclusion

The positive effects of PGPR on plant health have been well documented in literature; however, the inherent complexity of PGPR interactions with the environment, native flora, and crop-specific conditions contributes to inconsistent outcomes [46, 47]. To address these challenges, contemporary research efforts increasingly focus on the use of specific combinations of PGPR [23, 48–50] with a wide spectrum of modes of action, that is sufficiently robust to counter environmental factors, [49, 51]. For instance, recent studies by Calvo et al. [52] have highlighted the efficacy of tailored PGPR blends in enhancing nutrient uptake and stress tolerance in various crop species. Contextualizing our findings alongside the impact of soil phosphate (P*i*) levels on the individual isolates sourced from the Breedt et al. [23] consortium, it becomes evident that soil P*i* concentration plays a pivotal role in shaping the ability of these individual isolates to influence wheat yield. Studies by Wang et al. [22], Adesemoye et al., [29], and Batool and Altaf [30] have similarly emphasized the importance of soil nutrient dynamics in modulating PGPR efficacy. This reinforces the need to consider soil P*i* levels as a critical factor in the design and execution of future PGPR optimization trials within wheat cultivation. Our results show that these isolates can effectively reduce P*i* application rates increasing fertilizer-use efficiency when compared to current commercial farming practices. However, it is essential to acknowledge the limitations of our study. Future research should prioritize evaluating phosphate solubilization at the final stages of vegetative growth and unraveling the mechanisms between PGPR, the host plant, and the microbiome to address the inconsistency in performance as observed in the current study. By delving deeper into these complexities, we can shed light on the underlying mechanisms that drive the observed seasonal variations, ultimately facilitating more precise and effective PGPR applications in agriculture.

## Data Availability

Not applicable.
